# Management of Lumbar Disc Herniation With Nerve Compression in a 21-Year-Old Male: A Case Report Exploring Multifactorial Causes of Disc Herniation

**DOI:** 10.7759/cureus.77916

**Published:** 2025-01-24

**Authors:** Porus Palluppetta, Constantino G Lambroussis, Arjun Dhillon, Nathan Nuengchana, Rahul Rajput, Osamah Baig, Sanjana D Nalla

**Affiliations:** 1 College of Medicine, Lake Erie College of Osteopathic Medicine, Erie, USA; 2 Family Medicine, Lake Erie College of Osteopathic Medicine, Elmira, USA; 3 Medical Education, Lake Erie College of Osteopathic Medicine, Erie, USA; 4 College of Medicine, University of New England College of Osteopathic Medicine, Biddeford, USA; 5 Surgery, Lake Erie College of Osteopathic Medicine, Erie, USA; 6 Obstetrics and Gynecology, Lifeline Medical Associates, Edison, USA

**Keywords:** compression, disc, herniation, lumbar, nerve

## Abstract

Lumbar disc herniation (LDH) is a major cause of low back pain traditionally associated with age-related degenerative changes of intervertebral discs in adults aged 30-50 years. Symptoms of LDH can include pain, sensory disturbances, or motor deficits depending on the degree of nerve root compression. This case report highlights a 21-year-old male college student with LDH and nerve root compression, complicated by a prior anterior cruciate ligament (ACL) injury and a sedentary lifestyle. Magnetic resonance imaging (MRI) findings revealed a left L4 nerve root compression and a central-to-right paracentral herniation affecting the right S1 nerve root. The assessment demonstrated significant pain and reduced range of motion (ROM) in hip flexion and extension. Physical therapy utilizing a multimodal approach emphasizing spinal stabilization, postural correction, and mobility exercises was implemented to enhance the patient’s function and recovery process. Over the course of treatment, notable improvements in ROM and pain levels were observed. Treatment successfully culminated in minimal pain by the patient’s final session. After physical therapy had concluded, and our patient resumed regular activities, he was able to achieve complete pain remission in approximately three months with ROM also returning to baseline. This case report underscores the multifactorial contributors to LDH in young adults, emphasizing the importance of a comprehensive approach to rehabilitation in managing spinal injuries for optimal outcomes.

## Introduction

Lumbar disc herniation (LDH) is a major cause of low back pain and radicular symptoms, often resulting in substantial physical and functional impairment. Traditionally, LDH is associated with age-related degenerative changes of intervertebral discs, predominantly in adults aged 30-50 years [1]. However, recent studies have shown an increased incidence of LDH among younger populations, attributable to various lifestyle and biomechanical factors. Contributing factors include repetitive physical strain, poor ergonomics, and an increasingly sedentary lifestyle brought on by prolonged sitting for study, work, or recreational screen use [2,3]. In younger individuals, habits consisting of extended desk work or improper posture could lead to possible alteration in spinal biomechanics, predisposing individuals to disc pathology such as LDH [2].

Mechanical stress caused by prior musculoskeletal injuries, such as anterior cruciate ligament (ACL) tears, can lead to gait abnormalities, pelvic tilts, and abnormal weight distribution [4]. These biomechanical changes increase stress on lumbar intervertebral discs, particularly the L4-L5 and L5-S1 segments, which are most vulnerable to herniation [5]. The relationship between lower extremity injuries and lumbar spine pathology highlights the interdependence of the musculoskeletal system. Compensatory mechanics following knee injuries further exacerbate spinal stress, increasing susceptibility to disc degeneration and herniation in active individuals [6].

In addition to mechanical factors, sedentary behavior is a significant risk factor for lumbar spine degeneration. Prolonged sitting has been shown to increase intradiscal pressure by about 30% compared to upright standing, and contribute to chronic degeneration of the intervertebral discs [7]. Over time, this can lead to the weakening of the annulus fibrosus and eventual herniation of the nucleus pulposus, resulting in pain, sensory disturbances, or motor deficits depending on the degree and location of nerve root involvement [4,7]. Disc herniation can present with a spectrum of clinical manifestations, ranging from localized back pain to more severe cases involving sciatica, paresthesias, or muscle weakness.

Management strategies for LDH often begin with conservative approaches [8]. Physical therapy has demonstrated efficacy in reducing pain, improving range of motion (ROM), and restoring function. Targeted rehabilitation programs focus on strengthening paraspinal muscles, enhancing core stability, and alleviating nerve root compression [8]. Studies highlight that structured physical therapy regimens can result in symptom remission, improved mobility, and a return to daily activities without the need for invasive interventions [9,10]. These outcomes underscore the importance of early diagnosis and individualized management to optimize patient recovery and prevent long-term complications. The following case report describes a 21-year-old male with LDH and nerve root compression, emphasizing the multifactorial causes, including prior ACL injury and sedentary lifestyle, and highlighting the successful conservative management approach.

## Case presentation

**Figure 1:**
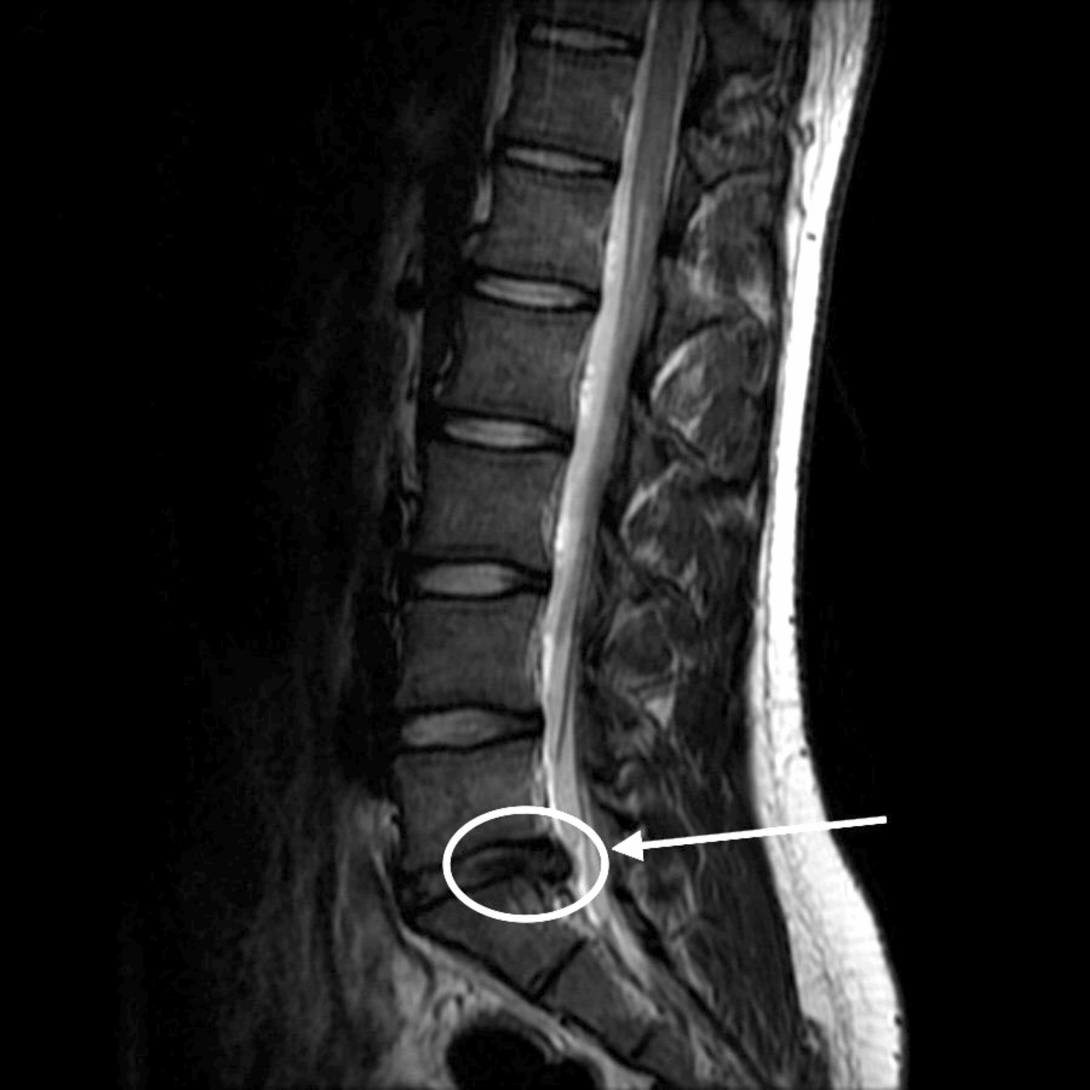
Sagittal T2-weighted MRI of the lumbar spine with a lateral herniation compressing the left L4 nerve root and a central-to-right paracentral herniation impacting the right S1 nerve root. There is a loss of lumbar lordosis (marked by circle).

**Figure 2:**
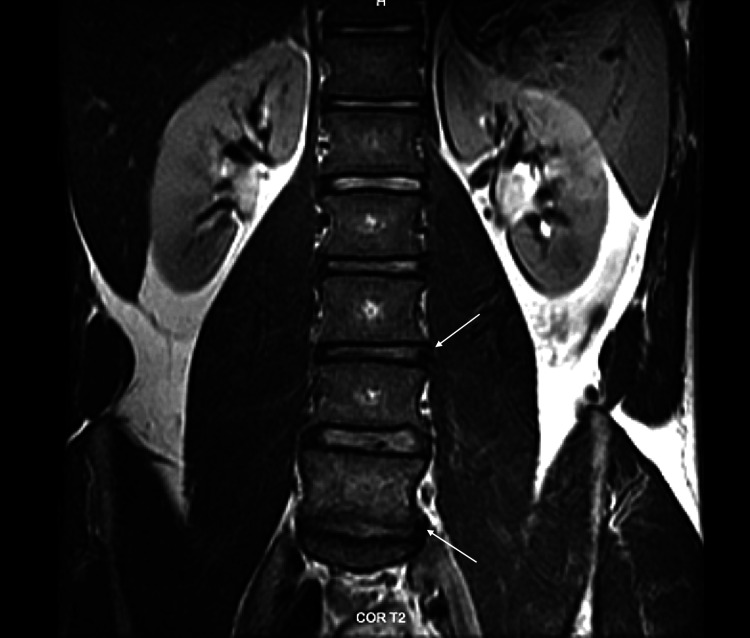
Coronal T2-weighted MRI of the lumbar spine demonstrating a lateral disc herniation compressing the left L4 nerve root and a central-to-right paracentral herniation impacting the right S1 nerve root (indicated by white arrows).

A 21-year-old male college student presented with significant lower back pain and radiating symptoms. Magnetic resonance imaging (MRI) revealed two disc herniations: a lateral herniation compressing the left L4 nerve root and a central-to-right paracentral herniation impacting the right S1 nerve root (Figures 1-2). A previous ACL injury two years prior, when our patient was 19 years old, likely altered the patient’s biomechanics, increasing lumbar spine stress. Prolonged sitting for study and work may have exacerbated spinal strain. Initial evaluation by physical therapy showed an elevated pain level rated at 9/10 with reduced hip flexion to 85/120 degrees with the knee flexed, and 65/90 degrees with the knee extended. These findings were consistent with acute pain and functional limitation. A multimodal approach, including manual therapy, stretching, and mobility exercises, was employed to address both ROM and pain management. An emphasis on ergonomics as well as frequent movement breaks was integral to the patient’s management plan, in conjunction with physical therapy sessions, in order to prevent the recurrence or worsening of symptoms.

After a month of therapy sessions, follow-up evaluation showed substantial mobility improvement with hip flexion with the knee flexed reaching 100/120 degrees, and pain level decreased to 6/10. With subsequent sessions, consistent progress was observed, with stable values in hip hyperextension at 25/30 degrees. By the final session, the patient reported minimal pain and had achieved a full normal active ROM. This allowed him to discontinue physical therapy and return to his daily activities and sports participation. Within three months, our patient had achieved complete pain remission with ROM also returning to baseline.

## Discussion

Our patient’s previous ACL injury likely contributed to an alteration in their gait. The altered gait’s influence towards placing additional stress on the lumbar spine likely contributed to additional pain and mobility limitation. ACL injuries can lead to biomechanical changes that impact lower back health, potentially contributing to lumbar disc issues [7]. Following knee surgery in 2019, the patient reported significant weight displacement onto the right leg, resulting in right hamstring tightness and reduced flexibility. This likely led to a posterior pelvic tilt impacting spinal alignment. Over time, a posterior pelvic tilt could reduce spinal lordosis putting additional strain on the intervertebral discs and muscles surrounding the spine [7]. Such alterations can predispose individuals to back pain and dysfunction by increasing shearing forces and reducing the spine’s natural shock absorption [2]. Sedentary behavior, particularly prolonged sitting, is known to increase intervertebral disc pressure and predispose individuals to lumbar disc degeneration and herniation [5]. Emphasizing ergonomics and frequent movement breaks was integral to our patient’s management plan, aligning with evidence linking prolonged sitting to lumbar disc pathology.

An LDH refers to when the nucleus pulposus, the gel-like central portion of an intervertebral disc, protrudes through a tear or weakness in the surrounding annulus fibrosus and may result in pain, sensory disturbances, and motor deficits [10]. The MRI findings of lateral herniation affecting the L4 nerve root and a central herniation compressing the S1 nerve root in our patient are consistent with the patient’s reported symptoms which included pain, sensory disturbance, and motor deficit. Addressing the specific nerve roots identified through MRI assisted in guided therapeutic approaches in our patient.

The ROM improvements and pain reduction achieved through conservative physical therapy interventions align with the documented benefits of conservative management for LDH [7]. The structured intervention led to significant clinical improvement, with the patient achieving full pain remission and a return to sports within three months, demonstrating the durability of the therapeutic approach [8].

## Conclusions

This case underscores the multifactorial LDH in young adults, where prior injuries, sedentary behavior, and poor ergonomics converge as risk factors. A structured physical therapy protocol focused on ROM enhancement, pain reduction, and functional recovery, provided effective symptom relief and mobility restoration. Such comprehensive management, combined with lifestyle modifications, can promote favorable outcomes and mitigate recurrence risks. Future research should focus on the long-term impacts of sedentary behavior and rehabilitative strategies for young adults with lumbar disc pathology.
